# Occupational Exposure to Needle Stick and Sharp Injuries and Postexposure Prophylaxis Utilization Among Healthcare Professionals in Southwest Ethiopia

**DOI:** 10.1155/cjid/3792442

**Published:** 2025-02-27

**Authors:** Tewodros Yosef, Adane Asefa, Hailemariam Amsalu, Melsew Setegn Alie, Aklilu Habte, Zemachu Ashuro, Aragaw Tesfaw, Nigusie Shifera

**Affiliations:** ^1^Department of Public Health, Mizan-Tepi University, Mizan Teferi, Ethiopia; ^2^Department of Population Health, Deakin University, Waurn Ponds, Victoria, Australia; ^3^Department of Biomedical Sciences, Mizan-Tepi University, Mizan Teferi, Ethiopia; ^4^Department of Public Health, Wachemo University, Hosanna, Ethiopia; ^5^Department of Environmental Health, Dilla University, Dilla, Ethiopia; ^6^Department of Public Health, Debre Tabor University, Debre Tabor, Ethiopia

**Keywords:** Ethiopia, healthcare professionals, needle stick injuries, postexposure prophylaxis, sharp injuries

## Abstract

**Background:** Occupational needle stick and sharp injuries (NSSIs) are significant public health concerns in the healthcare systems of developing countries. In Ethiopia, healthcare facilities commonly underreport these incidents. Therefore, this study aimed to assess occupational exposure to NSSIs and utilization of postexposure prophylaxis (PEP) among healthcare professionals at Mizan-Tepi University Teaching Hospital (MTUTH) in southwest Ethiopia.

**Methods:** A cross-sectional study was conducted among 196 healthcare professionals from September 1 to 15, 2021, at the MTUTH in southwest Ethiopia. The data were collected using a self-administered questionnaire. SPSS Version 21 was used for the data entry and analysis. Logistic regression was employed to identify the factors associated with the dependent variable, with significance set at *p* < 0.05.

**Results:** The magnitude of NSSIs was 18.9% (*n* = 37/196; 95% CI 13.4%, 24.4%). The proportion of injured participants who underwent PEP was 43.2% (*n* = 16/37). Being married [AOR = 2.89, 95% CI (1.11, 7.48)] and not undergoing infection prevention (IP) training [AOR = 4.32, 95% CI (1.40, 13.4)] were associated with an increased likelihood of NSSIs. Conversely, having good knowledge of IP [AOR = 0.17, 95% CI (0.07, 0.42)] was linked to a decreased likelihood of NSSIs.

**Conclusion:** Approximately one in five healthcare professionals experienced NSSIs. Factors associated with NSSIs include being married, receiving IP training, and possessing knowledge of IP. These findings underscore the importance of comprehensive IP training to enhance awareness of IP. Such interventions are critical for minimizing NSSIs and ensuring the safety of healthcare personnel.

## 1. Introduction

Needle stick and sharp injuries (NSSIs) are work-related accidental body punctures sustained in connection with the use of devices such as needles, knives, ampoules, and lancets used in healthcare settings and pose an occupational hazard to healthcare workers (HCWs) during the process of medical treatment [1, 2]. NSSIs pose significant risks to HCWs due to their connection with standards of practice [3, 4]. They are acknowledged as one of the foremost serious hazards for HCWs worldwide [5, 6]. NSSIs are accountable for about 40% of Hepatitis B and Hepatitis C infections, as well as 2.5% of HIV infections, among HCWs globally [6].

Contact with blood and body fluids is a major concern for healthcare providers through sharp instruments as part of their duty [7] and poses a major risk of transmitting infections including human immunodeficiency virus (HIV), hepatitis B virus (HBV) and hepatitis C virus (HCV) [8–14]. Occupational accidents involving needle sticks and sharp objects are a leading cause of bloodborne pathogen infections among HCWs [15–17], and every day, they are exposed to deadly pathogens through contaminated needles and other sharp injuries [18, 19].

For HCWs worldwide, the attributable proportions for percutaneous occupational exposure (OE) to HBV, HCV, and HIV are 37%, 39%, and 4.4%, respectively [20]. Occupational HIV exposure among HCWs is a growing global public health concern [21]. Postexposure prophylaxis (PEP) is a short-term antiretroviral treatment that reduces the risk of viral infection after exposure to infected blood or body fluids [22, 23]. Timely PEP following exposure to high-risk fluids at work significantly lowers HIV transmission rates [23].

Bouya et al.'s systematic review and meta-analysis (87 studies, 50,916 HCWs, 31 countries) reported a global 1-year pooled prevalence of NSSIs at 44.5% (95% CI: 35.7–53.2). The highest prevalence was in Southeast Asia (58.2%) and the Eastern Mediterranean Region (53.5%), while Africa reported 41.7% [3]. Injuries from needle sticks and sharp objects among HCWs average four per person per year in sub-Saharan Africa [24]. The magnitude of NSSIs in Ethiopia is ranging from 18.7% to 62.8% [8, 17–19, 25–32].

Occupational health and safety risks are high in organizations that provide healthcare, particularly in hospitals [33]. NSSIs are very dangerous for HCWs working in all areas of healthcare [34, 35]. The burden of NSSIs is more common in developing than in developed countries [34]. OE to NSSIs remains a major public health concern in the healthcare systems of developing countries [36]. There is significant underreporting of NSSIs worldwide, with the actual incidence of NSSIs being much higher than that reported [37]. In Ethiopia, OE to NSSIs is frequently underreported in many healthcare facilities [30], and there has been limited routine utilization of PEP by workers [22]. Therefore, this study aimed to assess the OE to NSSIs and utilization of PEP among healthcare professionals at MTUTH in southwest Ethiopia.

## 2. Methods

### 2.1. Study Design, Setting, and Period

A cross-sectional study took place at MTUTH in southwest Ethiopia, from September 1 to 15, 2021. Mizan-Tepi University Teaching Hospital (MTUTH), established in 1986, is one of the oldest and busiest hospitals in southwest Ethiopia. Located in the Bench Sheko Zone, 585 km southwest of Addis Ababa, it serves a catchment population of 2.75 million people. The hospital provides specialized services in obstetrics/gynecology, pediatrics, surgery, and general medicine and handles referrals from the Gambella and southwest Ethiopia regions. It is staffed by approximately 449 health professionals.

### 2.2. Populations

During the study period at MTUTH, all healthcare professionals constituted the source population, while randomly selected healthcare professionals comprised the study population. Only those healthcare professionals on duty during the specified period were included, while those not on active duty or unwilling to participate were excluded. The details were better outlined in a prior publication [38].

### 2.3. Sample Size Determination and Sampling Technique

The sample size was calculated using a single population proportion formula, assuming a NSSI rate of 50% among health professionals, as there were no prior studies in the area. With a 95% confidence level and a 5% margin of error, the initial sample size was determined to be 384:

(1) $$\begin{array}{c} n=\frac{{\left(Z\;\alpha /2\right)}^{2}p\left(1-p\right)}{d^{2}}=\frac{{\left(1.96\right)}^{2}0.5\left(1-0.5\right)}{{\left(0.05\right)}^{2}}=384. \end{array}$$

Since the total population is less than 10,000, the corrected sample size formula was applied. *N* = *n*/(1 + (*n*/*N*)) = 384/(1 + (384/333)) = 179. After incorporating a 10% allowance for nonresponse compensation, the final sample size was determined to be 197. Sampling units were chosen employing a systematic random sampling technique. The details were better outlined in a prior publication [38].

### 2.4. Study Variables and Measurements

The study considered NSSIs as the dependent variable. The independent variables included sociodemographic characteristics such as sex, age, marital status, professional qualification, working department, work experience, and working hours per day, as well as occupational factors such as needle recap practices, waste segregation practices, availability of sufficient personal protective equipment (PPE), availability of infection prevention (IP) manuals, availability of IP training, and knowledge about IP.

NSSIs were evaluated via self-reported instances of skin puncture by needles or sharp objects previously exposed to blood, tissue, or bodily fluids [28].

For the variable “knowledge about IP,” the measurement details were better outlined in a prior publication [38].

HIV PEP is indeed an antiretroviral therapy given to HCWs or individuals who have experienced OE to HIV. This exposure could occur through percutaneous injury (such as a needle stick or cut with a sharp object), contact with mucous membranes, nonintact skin (like chapped or abraded skin), or prolonged contact with skin involving a large area [23].

### 2.5. Data Collection Instrument and Procedure

The authors developed a structured questionnaire in English, tailored to the study's objectives. The data were collected using a self-administered questionnaire. This questionnaire covered inquiries regarding health facility–related characteristics, exposures to needle stick and sharps, history of PEP, and sociodemographic details. Before data collection, a pretest was conducted on 5% of the sample size at the hospital other than the study setting. Data collectors and supervisors underwent training on the objectives, procedures, and how to address any ambiguities in the questionnaire.

### 2.6. Statistical Analysis

The data were entered and analyzed using SPSS Version 21. Descriptive statistics were employed, and the study results were articulated through frequencies and percentages, showcased via tables. Bivariate and multivariable logistic regression analyses were utilized to pinpoint independent variables associated with the dependent variables. Independent variables with a *p* value < 0.25 in bivariate logistic regression were incorporated into the multivariable logistic regression model. Significance was determined at a *p* value of < 0.05.

### 2.7. Ethics Approval and Consent to Participate

Ethical clearance for the study was granted by the Research Review Committee of Mizan-Tepi University, College of Medicine and Health Sciences (PGC/069/2021). Study participants were briefed on the study's purpose, their right to decline participation, and the assurance of anonymity and confidentiality of their information, in accordance with the Declaration of Helsinki. Written informed consent was obtained from all participating individuals.

## 3. Results

### 3.1. Sociodemographic Characteristics

In the survey, 37.8% of respondents were married. The majority (92.9%) had less than 5 years of working experience, while nearly one-third (28.1%) worked eight or more hours per day (Table 1).

### 3.2. Health Facility–Related Factors

Most participants, specifically 90.3%, reported recapping needles, and 182 participants, constituting 90.9% of the total, reported segregating waste. Additionally, 159 participants, representing 81.1% of the sample, reported taking IP training (Table 2).

### 3.3. Magnitude of NSSIs and PEP

**Figure 1:**
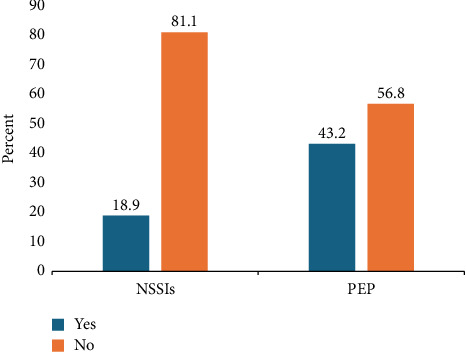
Prevalence of NSSIs and PEP among participants at MTUTH in southwest Ethiopia.

Among participants, the prevalence of NSSIs was 18.9% (*n* = 37/196, 95% CI 13.4%, 24.4%). Additionally, the proportion of injured participants who received PEP was 43.2% (*n* = 16/37) (Figure 1).

### 3.4. Factors Associated With NSSIs

After adjusting for confounding variables, being married [AOR = 2.89, 95% CI (1.11, 7.48)] and not taking IP training [AOR = 4.32, 95% CI (1.40, 13.4)] were linked to an increased likelihood of NSSIs. Conversely, having good knowledge of IP [AOR = 0.17, 95% CI (0.07, 0.42)] was linked to a decreased likelihood of NSSIs (Table 3).

## 4. Discussion

NSSIs are recognized as a primary occupational hazard for HCWs globally [5, 6]. This study aimed to assess the OE to NSSIs and utilization of PEP among healthcare professionals at MTUTH in southwest Ethiopia. The prevalence of NSSIs was 18.9% (37 out of 196), with a 95% confidence interval of 13.4%–24.4%. Among the injured participants, 43.2% (16 out of 37) received PEP. The study also identified marital status, IP training, and knowledge of IP as factors associated with NSSIs.

The prevalence of NSSIs in this study aligned with the 18.7% prevalence in Awi zone, Ethiopia [27]. However, it was lower than 25.9% central zone of Tigray [26], 29.5% in Northwestern [28], 31% in Bahir Dar [19], 31% in Hawassa town [31], 32.8% in Northeast [25], 34.5% in Dessie town [29], 36.2% in Addis Ababa [32], and 38.3% in Bale zone [30] studies in Ethiopia. The variation observed between this and other studies could be the sociodemographic characteristics, the sample size, and the methodology used to ascertain the outcome. Despite all studies using a 12-month prevalence to assess NSSIs, discrepancies, such as focusing solely on nurses [32], and variations in study settings—some conducted in general hospitals, others in referral hospitals, and still others in specialized hospitals—contribute to inconsistencies in the findings.

In this study, a significant association was found between marital status and NSSIs. Married participants exhibited 2.9 times higher odds of developing NSSIs compared to unmarried participants. This finding contradicts a study from Eastern Ethiopia [16], suggesting that needle stick injuries are more common among unmarried individuals. Married individuals may demonstrate a stronger sense of responsibility and adherence to safety guidelines [16].

Taking IP training was important for preventing infection. Participants who did not take IP training had 4.3 times higher odds of developing NSSIs than those who had IP training. This finding was supported by other studies conducted elsewhere [17, 25, 29, 39]. Infection control training indirectly reduces NSSIs by emphasizing sharps awareness, universal precautions, and improved decision-making among HCWs [40]. While not the sole focus, such training promotes a safer work environment by instilling awareness and responsible sharps handling. However, a comprehensive approach to reducing NSSIs requires a combination of strategies, including safety devices, engineering controls, regular training, and infection control practices [41]. The hospital training unit reports that while there is no formalized schedule for IP training, all health professionals employed for at least 1 year receive annual training whenever IP practices are updated. New employees receive training upon hire.

Knowledge of IP was significantly associated with NSSIs. Participants with good knowledge of IP had 83% less odds of developing NSSIs than those with poor knowledge of IP. This finding was supported by a study conducted in Egypt [42]. Comprehending IP is essential in mitigating NSSIs among HCWs. A comprehensive understanding promotes safe practices, encompassing the use of safe sharp devices and proper needle disposal. Increased awareness supports effective decision-making in identifying and responding to high-risk situations. Research indicates a significant correlation between IP knowledge and reduced NSSI rates [43]. Nonetheless, implementing safety measures such as safety-engineered sharps devices remains equally imperative.

In this study, the proportion of PEP among injured participants was 43.2%, 95% CI (27.1%, 60.5%). This finding was consistent with 48.6% at the University of Gondar Comprehensive Specialized Hospital in Ethiopia [44]. This finding was higher than 16.1% at St. Peter's specialized hospital [21] and 19.6% at Mekelle town government health institution [45] studies in Ethiopia. However, it was lower than 68.4% at health centers in the Harari region [23] and 71.7% at Hiwot Fana Specialized University Hospital [22] studies in Ethiopia. The variations in observed differences between studies can be attributed to differences in the denominator used for calculating the PEP rate. Some studies, like one referenced [21], use the total sample size as the denominator, while others, like this study, employ the number of NSSIs as the denominator. The limitations of this study include its cross-sectional design, which limits the ability to establish causality between variables. Data collection via self-administered structured questionnaires could lead to response bias or underreporting of sensitive information.

## 5. Conclusion

Approximately one in five healthcare professionals experienced NSSIs. Factors associated with NSSIs include marital status, receiving IP training, and possessing knowledge of IP. These findings underscore the importance of comprehensive IP training to enhance awareness of IP. Such interventions are critical for minimizing NSSIs and ensuring the safety of healthcare personnel.

## Figures and Tables

**Table 1:** Sociodemographic characteristics of the participants at MTUTH in southwest Ethiopia.

Variables	Categories	Frequency	Percent
Gender	Male	114	58.2
	Female	82	41.8

Age	< 29 years	137	69.9
	≥ 29 years	59	30.1

Marital status	Unmarried	122	62.2
	Married	74	37.8

Professional qualifications	GP/specialists	43	21.9
	^#^Others	153	78.1

Working departments	Outpatient	51	26
	Ward	93	47.4
	Laboratory	10	5.1
	Emergency	37	18.9
	Others	5	2.6

Work experience	< 5 years	182	92.9
	≥ 5 years	14	7.1

Working hours per day	≤ 8 h	141	71.9
	> 8 h	55	28.1

^#^Others: health officers, midwives, nurses, X-ray technicians, pharmacists, and medical laboratory technicians.

**Table 2:** Health facility-related factors of the participants at MTUTH in southwest Ethiopia.

Variables	Categories	Frequency	Percent
Recap of needles	Yes	181	90.3
	No	15	7.7

Waste segregation	Yes	182	90.9
	No	14	9.1

Availability of sufficient PPE in the work area	Present	119	60.7
	Absent	77	39.3

Infection prevention manual at work	Present	148	75.5
	Absent	48	24.5

Taking infection prevention training	Yes	159	81.1
	No	37	18.9

Knowledge of infection prevention	Poor	55	28.1
	Good	141	71.9

**Table 3:** Factors associated with NSSIs among participants at MTUTH in southwest Ethiopia.

Variables	Categories	NSSIs		COR (95% CI)	AOR (95% CI)	*p* value
		Yes	No			
Marital status	Married	18	52	1.95 (0.94, 4.02)⁣^∗^	2.89 (1.11, 7.48)	0.029
	Not married	19	107	1	1	

Recapping needles	Yes	16	46	0.79 (0.64, 2.32)⁣^∗^	0.52 (0.09, 3.04)	0.468
	No	11	113	1	1	

Segregating wastes	Yes	31	147	2.37 (0.83, 8.40)⁣^∗^	1.52 (0.31, 7.69)	0.600
	No	6	12	1	1	

Taking IP training	Yes	18	141	1	1	
	No	19	18	8.27 (3.68, 18.6)⁣^∗∗^	4.32 (1.40, 13.4)	0.011

Sufficient PPE in the work area	Present	15	104	1	1	
	Absent	22	55	2.77 (1.33, 5.77)⁣^∗∗^	1.75 (0.65, 4.68)	0.268

IP manual at work	Present	20	128	1	1	
	Absent	17	31	3.51 (1.65, 7.48)⁣^∗∗^	1.04 (0.36, 2.99)	0.815

Knowledge of IP	Poor	25	30	1	1	
	Good	12	129	0.11 (0.05, 0.25)⁣^∗∗^	0.17 (0.07, 0.42)	< 0.001

Abbreviations: AOR, adjusted odds ratio; CI, confidence Interval; COR, crude odds ratio.

⁣^∗^*p* value < 0.25.

⁣^∗∗^*p* value < 0.05.

## Data Availability

The dataset is handled by the corresponding author and can be provided upon request.

## Nomenclature

AOR: Adjusted odds ratio
CI: Confidence interval
COR: Crude odds ratio
GP: General practitioner
IP: Infection prevention
NSSIs: Needle stick and sharp injuries
PEP: Postexposure prophylaxis
SPSS: Statistical Package for the Social Sciences
SD: Standard deviation
